# Dissociation Between Incubation of Cocaine Craving and Anxiety-Related Behaviors After Continuous and Intermittent Access Self-Administration

**DOI:** 10.3389/fnins.2021.824741

**Published:** 2022-02-07

**Authors:** Celine Nicolas, Trinity I. Russell, Yavin Shaham, Satoshi Ikemoto

**Affiliations:** Behavioral Neuroscience Branch, Intramural Research Program, National Institute on Drug Abuse, Baltimore, MD, United States

**Keywords:** incubation of craving, anxiety-related behaviors, cocaine, intermittent self-administration, novelty-induced hypophagia

## Abstract

Studies using either continuous or intermittent access cocaine self-administration procedures showed that cocaine seeking increases during abstinence (incubation of cocaine craving), and that this effect is higher after intermittent cocaine access. Other studies showed that cocaine abstinence is characterized by the emergence of stress- and anxiety-related states which were hypothesized to increase relapse vulnerability. We examined whether incubation of cocaine craving and anxiety-related behaviors are correlated and whether intermittent cocaine self-administration would potentiate these behaviors during abstinence. Male rats self-administered cocaine either continuously (6 h/day) or intermittently (5 min ON, 25 min OFF × 12) for 14 days, followed by relapse tests after 1 or 21 abstinence days. A group of rats that self-administered saline served as a control. Anxiety-related behaviors were measured on the same abstinence days, using the novelty induced-hypophagia test. Finally, motivation for cocaine was measured using a progressive ratio reinforcement schedule. Lever-presses after 21 abstinence days were higher than after 1 day and this incubation effect was higher in the intermittent access group. Progressive ratio responding was also higher after intermittent cocaine access. Intermittent and continuous cocaine access did not induce anxiety-like responses in the novelty-induced hypophagia test after 1 or 21 abstinence days. Independent of the access condition, incubation of cocaine seeking was not correlated with the novelty-induced hypophagia measures. Results suggest that cocaine-induced anxiety-related states during protracted abstinence do not contribute to incubation of cocaine craving. However, this conclusion is tentative because we used a single anxiety-related measure and did not test female rats.

## Introduction

Persistent high rate of relapse is a main characteristic of drug addiction and one of the major obstacles in drug addiction treatment (Hunt et al., 1971; Sinha, 2011). In humans, relapse can be triggered by exposure to cues and context associated with drug use (Wikler, 1973; O’Brien et al., 1992). In rats with continuous cocaine self-administration history, non-reinforced cocaine seeking in the presence of drug-associated cues and contexts progressively increases during forced abstinence (Neisewander et al., 2000; Grimm et al., 2001; Chauvet et al., 2012), a phenomenon termed incubation of cocaine craving (Lu et al., 2004; Pickens et al., 2011). These studies led to a translational study showing incubation of the response to cocaine cues in humans (Parvaz et al., 2016). Recently, we reported that incubation of cocaine craving in rats is potentiated after intermittent access cocaine self-administration (Nicolas et al., 2019), a procedure that models intermittent binge cocaine intake in human drug users (Zimmer et al., 2011; Allain et al., 2017).

In humans, abstinence from chronic cocaine use is characterized by the emergence of negative emotional states such as anxiety, stress, agitation, and depression (Gawin and Ellinwood, 1989; Fox et al., 2005). These abstinence-related negative emotional states are associated with increased relapse vulnerability (Kampman et al., 2006). In agreement, several rat studies showed increased anxiogenic-like responses (measured by the elevated plus maze, defensive burying, and dark/light tests) 2 days and 28 days after withdrawal from repeated non-contingent cocaine injections (Sarnyai et al., 1995; Basso et al., 1999; Georgiou et al., 2016). Similarly, time of burying behavior was increased 2 days after continuous limited-access cocaine self-administration (2-h/day) (Buffalari et al., 2012). Additionally, increased burying behavior was observed during both early (1 day) and late (14 and 42 days) abstinence after continuous long-access cocaine self-administration (6-h/day) (Aujla et al., 2008). Furthermore, chronic oral (30 or 60 days) and intravenous binge cocaine self-administration (12–48-h free access) increase startle response (an anxiogenic response) and ultrasonic distress vocalizations during early abstinence (Barros and Miczek, 1996; Mutschler and Miczek, 1998; Mutschler et al., 2001). Additionally, exposure to contextual cues associated with non-contingent cocaine injections elicit an anxiogenic-like response in the elevated plus-maze test (DeVries and Pert, 1998). Finally, two studies using the novelty-induced hypophagia test (Dulawa et al., 2004) showed that paternal but not maternal cocaine self-administration increases anxiety-related behaviors in male, but not female, offspring (White et al., 2016; Fant et al., 2019).

Together, these clinical and preclinical studies suggest that exposure to cocaine-associated cues and anxiety-related states during abstinence contribute to relapse vulnerability. However, the link between cocaine craving and anxiety-related behaviors during abstinence is unknown. Therefore, in the present study, we examined whether incubation of cocaine craving and anxiety-related states are correlated and whether intermittent cocaine self-administration would potentiate these behaviors during abstinence. In the study described below, we trained rats to self-administer cocaine for 6-h per day under either intermittent or continuous access and tested them repeatedly on abstinence days 1 and 21 for cocaine seeking and anxiety-like responses, using the novelty-induced hypophagia test (Dulawa et al., 2004; Bechtholt et al., 2007). We also measured on abstinence day 22 the motivation to self-administer cocaine using a progressive ratio reinforcement schedule (Richardson and Roberts, 1996).

## Materials and Methods

### Subjects

The subjects were 29 male Sprague-Dawley rats (Charles River) weighing 300–400 g at the time of surgery. Rats were housed 2/cage for 1–3 weeks before surgery, and individually after surgery. They were maintained on a reverse 12-h light–dark cycle (lights off at 8:00 A.M.). All rats had free access to chow and water in their home cage and free access to water during the self-administration sessions. Procedures were approved by the ACUC of NIDA-IRP and were in accordance with the guide for the care and use of laboratory animals (National Research Council [NRC], 2011).

### Surgery

The rats were anesthetized with isoflurane gas (5% induction, 2–3% maintenance) and a Silastic catheter was inserted into the jugular vein. The distal end of the tubing was passed subcutaneously to the mid-scapular region and the proximal end was fixed to a modified 22-gauge cannula encased in dental cement anchored to a polypropylene mesh (Caprioli et al., 2015, 2017). The mesh was placed on the back in the mid-scapular region. After the surgery, the rats were injected with ketoprofen (4.5 mg/kg s.c) to relieve pain and were given 5–7 days of recovery prior to the start of self-administration training. During the recovery and training phases, the catheters were flushed every 24–48 h with sterile saline containing gentamicin (4.25 mg/ml).

### Cocaine Self-Administration

**FIGURE 1:**
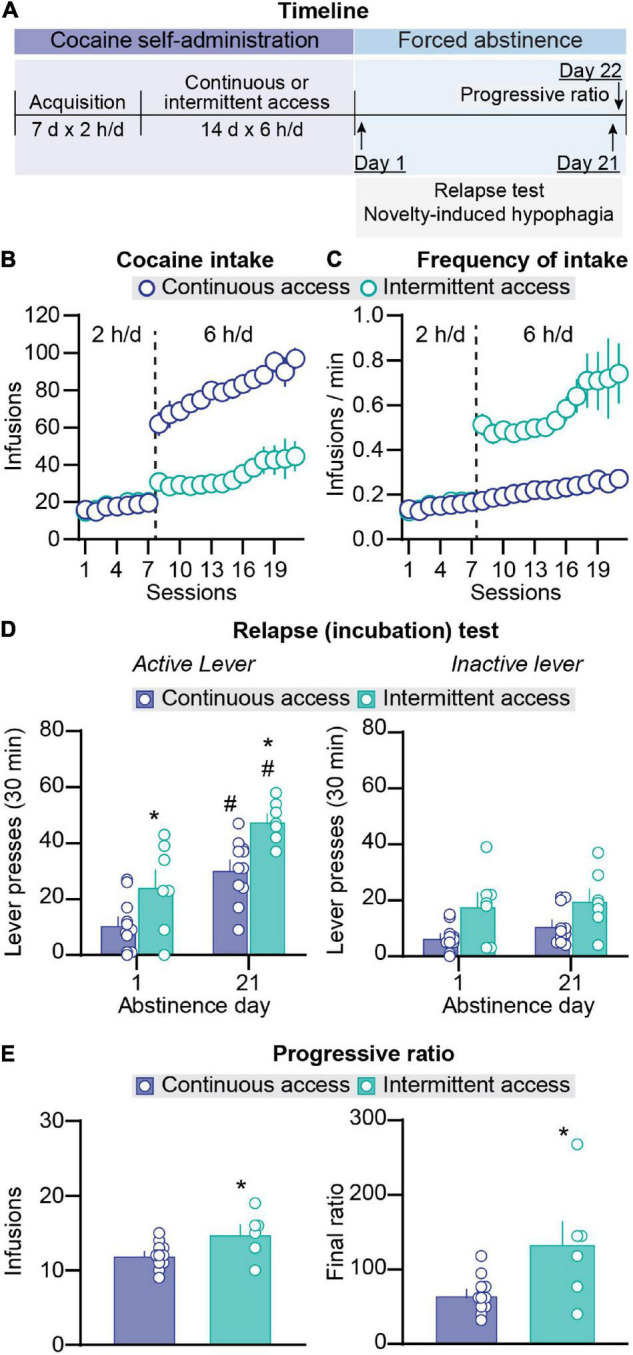
Effect of intermittent and continuous access cocaine self-administration on incubation of craving and progressive ratio reinforcement schedule responding. **(A)** Timeline of the experiment. **(B)** Total cocaine intake. Mean ± SEM number of infusions per session (continuous access *n* = 11, intermittent access *n* = 7). **(C)** Frequency of intake. Mean ± SEM number of infusions per min of cocaine access per session. Left side in all graphs (sessions 1–7): 2-h continuous access self-administration acquisition sessions; right side in all graphs (sessions 8–21): 6-h continuous or intermittent self-administration sessions (continuous access *n* = 11, intermittent access *n* = 7). **(D)** Relapse (incubation) test. Mean ± SEM number of active (left) and inactive (right) lever presses per session (continuous access *n* = 11, intermittent access *n* = 7 for both day 2 and day 21). **(E)** Progressive ratio reinforcement schedule. Mean ± SEM cocaine infusions (left) and final ratio completed (right) and during a single session in which lever presses were reinforced under a progressive ratio reinforcement schedule on abstinence day 22 (continuous access *n* = 11 intermittent access *n* = 6). #Different from day 1 within each schedule, *p* < 0.05. *Different from continuous access, *p* < 0.05.

Eighteen rats were trained to self-administer cocaine (Figure 1A) in operant conditioning chambers equipped with two levers, two cue lights above the levers, a tone, and a houselight. Each session started by inserting the two levers and illuminating the houselight. Pressing on the active lever delivered a cocaine infusion (0.1 ml/3.5 s; 0.75 mg/kg/infusion) and a compound tone-light cue for 3.5 s, followed by a 3.5-s timeout during which lever pressing was not reinforced. Pressing on the inactive lever had no programmed consequence. The rats were first trained to self-administer cocaine on a fixed-ratio 1 (FR1) schedule over 7 days for 2-h/day (max infusions = 20). Next, the rats self-administered cocaine either continuously or intermittently for 6-h/day for 14 days. In the continuous access condition, the rats had free access to cocaine during the daily sessions. In the intermittent access condition, the rats had access to cocaine during 12 × 5 min ON periods that were separated by 25-min OFF periods (Zimmer et al., 2011), corresponding to 60-min of cocaine access during the 6-h daily session. At the onset of each 5-min ON period, the lever was extended and the houselight was turned on; at the end of the 5-min access period, the active lever retracted and the houselight was turned off. Control rats (*n* = 11) received saline infusions according to a yoked procedure. Control rats received a saline infusion each time the paired cocaine rat self-administered a cocaine infusion. Active and inactive lever presses by control yoked rats were recorded but had no programmed consequence. Paired cocaine rats were counterbalanced between rats self-administered cocaine continuously (*n* = 6) or intermittently (*n* = 5).

### Drugs

(-)-Cocaine-HCl (cocaine) was obtained from the NIDA Drug Supply Program via the NIDA-IRP pharmacy and dissolved in sterile saline.

### Abstinence Phase

During this phase, the rats were housed in the animal facility and were handled two times per week.

### Anxiety-Related Tests

The rats (*n* = 29) were tested for anxiety-related behaviors on abstinence days 1 and 21 using the novelty-induced hypophagia and defensive burying tests. In a counterbalanced order, one anxiety test was performed before and the other after the relapse test. In the section “Results,” we will only present the results from the novelty-induced hypophagia test for the reason described below. Specifically, there were no significant differences between the continuous access and the saline groups on abstinence days 1 and 21 for either burying time or immobility time in the defensive burying test. For the intermittent access group, immobility time (but not burying time) was significantly higher on abstinence day 21 but not day 1 (data not shown). However, in a subsequent validation experiment (Supplementary Figure 1), we unexpectedly found that under our experimental conditions, intermittent footshock induces an anxiolytic-like response in the defensive burying test (decreased burying time; Treit et al., 1981). This unexpected result makes it impossible to interpret the results from the defensive burying test within the context of the relationship between anxiety-like responses and incubation of cocaine craving. In contrast, intermittent footshock induced the expected potentiation of anxiety-like responses in the novelty-induced hypophagia test (decreased food consumption and increased latency to start eating, Supplementary Figure 1). Below we describe in detail the procedures for both tests.

### Novelty-Induced Hypophagia

This test evaluates novelty-induced reaction (i.e., anxiety) by examining the latency to initiate the intake of a familiar palatable food in a novel (anxiogenic) environment (Dulawa et al., 2004; Bechtholt et al., 2007; White et al., 2016). Three days before the test, the rats had access to 8 g of palatable food (Graham Cracker Crumbs) placed in a plastic dish in their homecage to avoid neophobia. On abstinence day 1 and 21 tests, the rats were given access for 15 min to the same palatable food in a brightly lit (500 lux) novel environment. On abstinence day 1, the novel environment was a plastic box with white walls and floor without bedding, and on abstinence day 21, the novel environment was a cage with transparent walls and a wire mesh floor. The cage was cleaned with 70% ethanol and distilled water between each rat. All the sessions were videotaped, and the latency to start eating the palatable food and the quantity of food consumed were measured by an experimenter blind to the experimental conditions. Two rats (one from the intermittent access group on test day 1 and one from the saline group on test day 21) did not eat the food during the test. For the data analysis of these rats, we assigned the latency to eat value of 15 min (the maximum test duration).

### Defensive Burying

This test evaluates response to a threatening or noxious object (i.e., shock probe) by examining two behavioral outcomes, the burying behavior and the time of immobility (Treit et al., 1981). The defensive burying procedure was performed in a clear polycarbonate cage with a 1 cm diameter hole located 7 cm from the base of one end of the cage to hold the shock probe. The cage contained fresh bedding to a depth of 5 cm with the light set to 150 lux. The shock probe was extended 6 cm into the cage and connected to a shock generator to deliver 1.5 mA scrambled shock when the probe was contacted. The rats were habituated to the apparatus in the absence of the shock probe for 1-h per day for 2 days before the tests. At the beginning of each 15-min test, the rats were placed on the end opposite the shock probe, facing the probe. The duration of shock depended on the speed of the reflex of each rat upon contact. The probe remained electrified for the duration of the session. Bedding was changed between each rat, and the cage and probe were cleaned with 70% ethanol and distilled water. All the sessions were videotaped, and behavioral responses, including time spent burying and time of immobility were measured by an experimenter blind to the experimental conditions.

### Relapse Tests

On test days, the rats were placed in the same chambers where they previously self-administered cocaine. The relapse test was performed approximately 2 h after the first anxiety test (∼10.30 am). The relapse tests on abstinence days 1 and 21 were conducted under extinction conditions in the presence of the drug-associated cues during a single 30-min session. The session began with the illumination of the houselight and the extension of the active and inactive levers. Active lever presses resulted in contingent presentations of the tone/light cue for 3.5-s, but not cocaine; inactive lever presses had no programmed consequence. The number of non-reinforced active lever presses is the operational measure of drug seeking in incubation studies (Shalev et al., 2002; Lu et al., 2004; Pickens et al., 2011; Venniro et al., 2016).

### Progressive Ratio Reinforcement Schedule

On abstinence day 22, the rats were tested under a progressive ratio reinforcement schedule, a measure of the reinforcing effects of drug and non-drug reinforcers (Richardson and Roberts, 1996). During the test session, the ratio of responses per cocaine infusions was increased according to the following sequence: 2, 4, 6, 9, 12, 15, 20, 25, 32, 40, 50, 62, 77, 95, 118, 145, 178, 219… (Richardson and Roberts, 1996). The final completed response ratio represents the “breaking point” value and the program was turned off when 30-min elapsed without the rat earning an infusion. One rat from the intermittent access group with non-patent catheter was excluded.

### Footshock Exposure

To validate that the defensive burying and novelty-induced hypophagia tests induce anxiety-related behaviors under our experimental conditions, we performed a control experiment in which the rats were exposed to aversive uncontrollable and unpredictable footshock before being tested in the two tests. The rats (*n* = 8) were placed in a chamber equipped with a grid floor through which electric shock was delivered and were exposed to 10 min of footshock stress (1 mA for 0.5 s repeated with a variable interval of 40 s). Control rats (*n* = 8) were placed in the same chamber but were not exposed to footshock. Two hours after the footshock procedure, the rats underwent the defensive burying and the novelty-induced hypophagia tests in a counterbalanced order. The tests were separated by 3 h. All the sessions were videotaped, and behavioral responses were measured by an experimenter blind to the experimental conditions. During the novelty-induced hypophagia test, four rats from the intermittent footshock group did not eat the food. For the data analysis of these rats, we assigned the latency to eat value of 15 min (the maximum test duration).

### Statistical Analyses

We analyzed the data with ANOVAs, using SPSS (GLM module). We followed up on significant main and interaction effects (*p* < 0.05) with Bonferroni *post hoc* test. Detailed statistical analyses are described in the section “Results.” Our multifactorial ANOVAs yielded multiple main and interaction effects. Thus, we only report significant effects that are critical for data interpretation (see Supplementary Table 1 for a complete statistical reporting).

## Results

### Effect of Intermittent Cocaine-Self-Administration on Incubation of Craving and Progressive Ratio Responding

#### Cocaine Self-Administration

During the acquisition phase in sessions 1–7 (2-h/day continuous access), there were no differences in the number of infusions and the frequency of infusions (infusions/min) between the rats that were assigned to continuous and intermittent cocaine self-administration (Figures 1B,C). The statistical results of the acquisition phase are described in Supplementary Table 1. During the training phase in sessions 8–21 (6-h/day continuous or intermittent access), escalation of cocaine intake over days was observed under both schedules. In addition, total daily cocaine intake was higher in the continuous-access condition whereas the infusion rate per minute was higher in the intermittent-access condition. The statistical analysis for number of daily cocaine infusions, which included the between-subjects factor of group (continuous, intermittent) and the within-subjects factor of session, showed significant effects of group (*F*_1_,_16_ = 52.8, *p* < 0.001) and session (*F*_13_,_208_ = 8.7, *p* < 0.0001) but no significant interaction (*p* > 0.05). The analysis of daily infusion rate (number of infusions per min) showed significant effects of group (*F*_1_,_16_ = 75.5, *p* < 0.0001) and session (*F*_13_,_208_ = 5.0, *p* < 0.0001) but no significant interaction (*p* > 0.05).

#### Relapse Tests

On abstinence days 1 and 21, we measured non-reinforced lever presses (cocaine seeking) in the presence of cues and context associated with cocaine self-administration during the training phase. Independent of the abstinence day, cocaine seeking was higher after intermittent access than after continuous access cocaine self-administration (Figure 1D). In addition, independent of the training schedule, cocaine seeking was higher on abstinence day 21 than on abstinence day 1 (incubation of cocaine craving). Finally, independent of the abstinence day, the number of inactive lever presses was higher after intermittent access than after continuous access (Figure 1D). The statistical analysis for number of lever presses, which included the between-subjects factor of group and the within-subjects factor of abstinence day (1, 21) and lever (active, inactive), showed significant effects of group (*F*_1_,_16_ = 16.39, *p* = 0.0009), abstinence day (*F*_1_,_16_ = 33.15, *p* < 0.0001), lever (*F*_1_,_16_ = 38.84, *p* < 0.0001), and abstinence day × lever interaction (*F*_1_,_16_ = 37.60, *p* < 0.0001). *Post hoc* analyses of number of active lever presses showed significant differences between abstinence day 1 and day 21 for both groups (*p* values < 0.05) and significant difference between the intermittent vs. continuous access groups on both abstinence day 1 and day 21 (*p* < 0.05), but no significant differences between active and inactive lever presses on abstinence day 1 for both groups and no difference between abstinence day 1 and day 21 for inactive lever presses for both groups (*p* values > 0.05).

#### Progressive Ratio

On abstinence day 22, we tested the rats for their motivation to self-administer cocaine using a progressive ratio reinforcement schedule. The number of infusions earned were higher after intermittent access than after continuous access (*t*_15_ = 2.5, *p* = 0.03; Figure 1E). In summary, in agreement with results from our previous study (Nicolas et al., 2019), we found that compared with continuous access cocaine self-administration, intermittent access cocaine self- administration increased cocaine seeking during both early and late abstinence. We also found that during late abstinence, progressive ratio responding was higher after intermittent access than after continuous access cocaine self-administration. These results replicate and extended previous reports (Zimmer et al., 2011, 2012; Kawa et al., 2016; Allain et al., 2017, 2018).

### Anxiety-Related Behaviors During Cocaine Abstinence

#### Novelty-Induced Hypophagia

**FIGURE 2:**
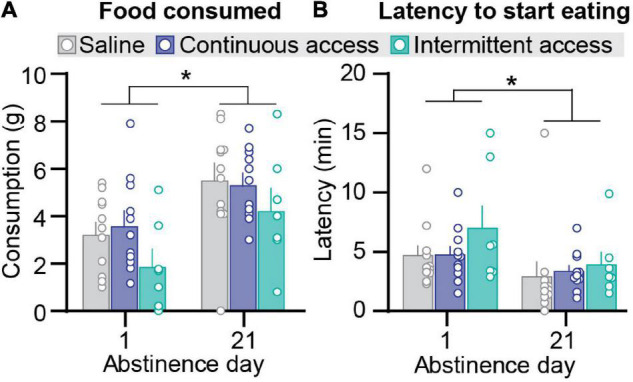
Effect of intermittent and continuous access cocaine self-administration on novelty-induced hypophagia. **(A)** Food consumed. Mean ± SEM grams of food consumed per session (saline *n* = 11, continuous access *n* = 11, intermittent access *n* = 7 for both day 1 and day 21). **(B)** Latency to start eating. Mean ± SEM min to start eating per session (saline *n* = 11, continuous access *n* = 11, intermittent access *n* = 7). *Different from Abstinence day 1, *p* < 0.05.

We assessed anxiety-related behavior in the novelty-induced hypophagia test using two established measures: amount of food consumed and latency to initiate feeding (Dulawa et al., 2004; Bechtholt et al., 2007). Intermittent or continuous cocaine self-administration had no significant effect on either measure (Figures 2A,B). On both abstinence day 1 and day 21, the intermittent and continuous cocaine access groups were not significantly different from the saline group for both the amount of food consumed and latency to initiate feeding. However, independent of the group condition, the amount of food consumed was higher on abstinence day 21 than on day 1, and the latency to initiate feeding was higher on abstinence day 1 than on day 21. The statistical analysis, which included the between-subjects factor of group (saline, cocaine continuous access, cocaine intermittent access) and test order (testing for novelty-induced hypophagia before or after the relapse test) and the within-subjects factor of abstinence day, showed a significant effect of abstinence day (*F*_1_,_23_ = 43.68, *p* < 0.0001 and *F*_1_,_23_ = 6.75, *p* < 0.05 for food consumption and latency, respectively) but no significant effects of group or interactions group and abstinence day or test order (*p* values > 0.05).

**FIGURE 3:**
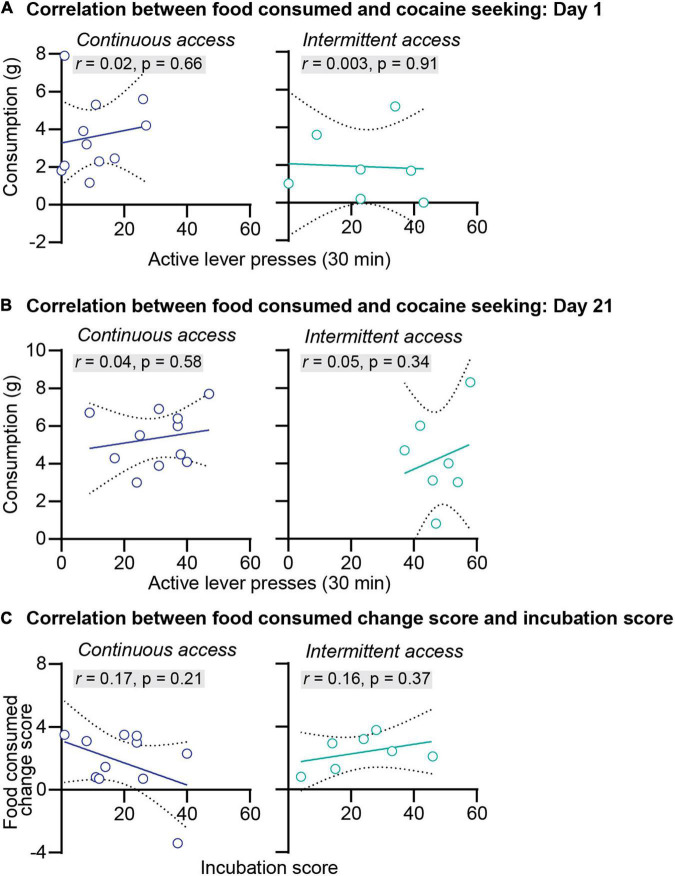
Correlations between the food consumed and incubation of cocaine craving. **(A)** Correlations between the amount of food consumed in grams and the number of active lever press during relapse test on abstinence day 1 after continuous (left) and intermittent (right) self-administration (continuous access *n* = 11, intermittent access *n* = 7). **(B)** Correlations between the amount of food consumed in grams and the number of active lever press during relapse test on abstinence day 21 after continuous (left) and intermittent (right) self-administration (continuous access *n* = 11, intermittent access *n* = 7). **(C)** Correlations between the food consumed change score (grams of food consumed on abstinence day 21 minus grams of food consumed on abstinence day 1) with the incubation score (active lever presses on abstinence day 21 minus active lever presses on day 1) after continuous (left) and intermittent (right) cocaine self-administration (continuous access *n* = 11, intermittent access *n* = 7).

**FIGURE 4:**
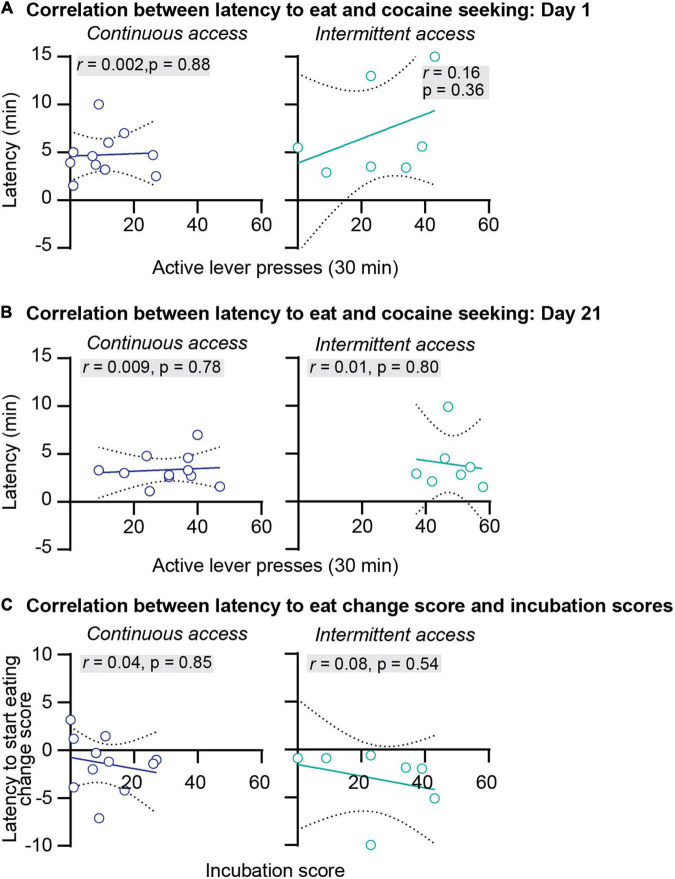
Correlations between the latency to start eating and incubation of cocaine craving. **(A)** Correlations between the latency to start eating in minutes and the number of active lever press during relapse test on abstinence day 1 after continuous (left) and intermittent (right) self-administration (continuous access *n* = 11, intermittent access *n* = 7). **(B)** Correlations between the latency to start eating in minutes and the number of active lever press during relapse test on abstinence day 21 after continuous (left) and intermittent (right) self-administration (continuous access *n* = 11, intermittent access *n* = 7). **(C)** Correlations between the latency to start eating change score (minutes to start eating on abstinence day 21 minus minutes to start eating on abstinence day 1) with the incubation score (active lever presses on abstinence day 21 minus active lever presses on day 1) after continuous (left) and intermittent (right) cocaine self-administration (continuous access *n* = 11, intermittent access *n* = 7).

We further examined the relationship between anxiety-related behaviors and incubation of cocaine craving. First, we correlated the food consumed or the latency to start eating with cocaine seeking at day 1 or day 21 of abstinence. The results of this analysis for the intermittent and continuous access group are presented in Figures 3A,B, 4A,B. This analysis showed no correlation between the food consumed or the latency to start eating with cocaine seeking on day 1 or day 21 (see Pearson *r* values and their *p* values in Figures 3A,B, 4A,B and Supplementary Table 1). Second, we examined the relationship between the change score of the anxiety-related measures (food consumed or latency to eat on day 21 value minus day 1 values) and the incubation score (active lever presses on abstinence day 21 minus active lever presses on abstinence day 1). This analysis also showed no significant correlations between the food consumed change score with the incubation score (Figure 3C) or between the latency to start eating change score with the incubation score (Figure 4C, see Pearson *r* values and their *p* values in Figures 3, 4 and Supplementary Table 1).

In summary, we found that continuous and intermittent cocaine self-administration did not induce anxiety-related states in the novelty-induced hypophagia test during either early or late abstinence. Additionally, no correlations were observed between the anxiety-related measures and cocaine seeking or incubation of cocaine craving. These results suggest that incubation of cocaine craving is independent of the emergence of anxiety-related states during abstinence.

### Effect of Intermittent Footshock-Induced on Anxiety-Like Behaviors in the Novelty-Induced Hypophagia and Defensive Burying Tests

We found no evidence that under our experimental conditions cocaine self-administration resulted in anxiety-like behaviors in the novelty-induced hypophagia test and the defensive burying test (see section “Materials and Methods”). Therefore, to verify that under experimental conditions, the two tests can detect anxiety-like states in rats, we expose the rats to intermittent footshock using parameters that induce physiological stress responses (increased peripheral corticosterone secretion and extrahypothalamic corticotropin-releasing factor neurotransmission) and reinstatement of drug seeking (Shaham et al., 2000; Mantsch et al., 2016). As expected, we found that intermittent footshock induced anxiety-like responses in the novelty-induced hypophagia test (Supplementary Figures 1A,B). Intermittent footshock significantly decreased food intake (*t*_14_ = 3.3, *p* = 0.005) and significantly increased latency to initiate food intake (*t*_14_ = 4.28, *p* = 0.0008). In contrast, unexpectedly, in the defensive burying test (Supplementary Figures 1C,D), intermittent footshock significantly decreased burying time (*t*_14_ = 2.8, *p* = 0.01) and had no significant effect on immobility time (*p* > 0.05). This result contrasts with general notion that burying behavior reflects anxiety-related states (Treit et al., 1981) that should be increased after intermittent footshock.

## Discussion

We examined whether incubation of cocaine craving and anxiety-related behaviors are correlated and whether intermittent cocaine self-administration would potentiate these behaviors during forced abstinence. As in previous studies, we found reliable incubation of cocaine craving after both continuous and intermittent cocaine self-administration and that this incubation was higher after intermittent drug access. In addition, and in agreement with previous reports, progressive ratio responding was higher after intermittent cocaine self-administration. In contrast, we found no evidence that intermittent or continuous cocaine self-administration induced anxiety-related behaviors in the novelty-induced hypophagia test during either early or late abstinence. Finally, independent of the cocaine access condition, incubation of cocaine craving was not correlated with the novelty-induced hypophagia measures. These data should be interpreted with caution because they rely on a single anxiety-related test. However, a conclusion from our study is that the emergence of anxiety- and stress-like states during protracted abstinence does not contribute to the emergence of incubation of cocaine seeking.

### Incubation of Craving and Progressive Ratio Responding After Intermittent and Continuous Access

Both continuous and intermittent cocaine self-administration induced incubation of cocaine craving after forced abstinence. These results confirm those from many previous studies using continuous access cocaine self-administration (Grimm et al., 2001; Wolf, 2016; Dong et al., 2017). Our results showing that cocaine seeking during the relapse tests was higher on abstinence day 1 and day 21 after intermittent access cocaine self-administration also confirm those from our recent study (Nicolas et al., 2019). These results, as well as our progressive ratio results showing higher final ratio after intermittent access cocaine self-administration, extend those from several studies reporting a strong motivation to seek and take cocaine, as assessed by resistance-to punishment, progressive ratio, economic-demand, and extinction-reinstatement procedures (Zimmer et al., 2012; Calipari et al., 2015; Kawa et al., 2016; Allain et al., 2018; Singer et al., 2018; Allain and Samaha, 2019; James et al., 2019; Nicolas et al., 2019).

However, our present and previous results contrast with those from a recent study showing similar incubation of cocaine craving after continuous vs. intermittent cocaine self-administration (Venniro et al., 2021). The reasons for the different results are unknown but may be due to procedural differences, including longer duration daily sessions (12 h per day in Venniro et al., 2018) and prior training to lever press for rewarding social interaction (Venniro et al., 2018) before cocaine self-administration in Venniro et al. (2021). Based on previous reports showing the protective effect of social reward on drug seeking and craving (Solinas et al., 2008; Venniro et al., 2018; Fredriksson et al., 2021), prior social interaction with a peer during the social self-administration phase may have decreased cocaine craving after intermittent access cocaine self-administration.

Finally, we found that intermittent cocaine access increased inactive lever presses during the relapse tests, the reasons for this effect, which we also observed in our previous study (Nicolas et al., 2019), are unknown. Increased inactive lever presses in extinction-reinstatement and incubation of craving studies likely reflects response generalization that commonly occurs when the operant response is not reinforced (Shalev et al., 2002). We speculate that this learning process is potentiated by intermittent cocaine self-administration.

### Lack of Emergence of Anxiety-Like Responses After Intermittent and Continuous Cocaine Access

We measured anxiety-related behaviors during abstinence after continuous or intermittent cocaine self-administration using the defensive burying and novelty-induced hypophagia tests. In the defensive burying test, intermittent but not continuous self-administration increased immobility time on abstinence day 21 but not day 1 without affecting burying behavior. However, these results are impossible to interpret because, unexpectedly, under our experimental conditions, intermittent footshock decreased probe burying time. Decreased burying time is the classical response to anxiolytic drugs of different drug classes in this test (Treit et al., 1981). These paradoxical results prevent any conclusions within the context of the relationship between anxiety-related behaviors and incubation of cocaine craving.

In the novelty-induced hypophagia test, we found no significant differences between the continuous or intermittent access groups vs. the saline control group for both the food consumed and the latency to start eating on either abstinence day 1 or 21. Our results contrast with those from previous studies reporting increased anxiety-related behaviors in the defensive burying, light-dark, elevated plus-maze, acoustic startle response, and ultrasonic vocalizations tests after non-contingent exposure to cocaine or cocaine self-administration (Sarnyai et al., 1995; Barros and Miczek, 1996; Mutschler and Miczek, 1998; Basso et al., 1999; Mutschler et al., 2001; Aujla et al., 2008; Buffalari et al., 2012; Georgiou et al., 2016; Nunes et al., 2019). What may account for these different results?

Beyond the use of different anxiety-related tests, methodological differences may account for the discrepant results between our study and previous cocaine self-administration studies. As in our study, Aujla et al. (2008) trained rats for 6 h per day but for a longer duration (22 days) than in our study (14 days). Mutschler and Miczek (1998) and Mutschler et al. (2001) trained their rats in a binge procedure in which rats are repeatedly given unlimited access to cocaine for 12, 16, and 48 h, resulting in very high cocaine intake. Thus, the higher cumulative cocaine intake in these studies may have led to reliable anxiety-like responses during abstinence. However, higher cumulative cocaine intake cannot explain the differences between the results of our study and studies that reported increased defensive burying on abstinence day 2 after limited access (2 h per day) cocaine self-administration training (Waters and See, 2011; Buffalari et al., 2012; Connelly et al., 2020). On the other hand, our negative findings agree with those from previous studies showing that different regimens of cocaine self-administration do not increase anxiety-related behaviors, as assessed in the open field, dark-light, and elevated plus-maze tests (Waters and See, 2011; Buffalari et al., 2012; Connelly et al., 2020).

Finally, a limitation of our study is the exclusive use of male rats. In this regard, both incubation of cocaine craving and anxiety-related behaviors were shown to be higher in female rats (Kerstetter et al., 2008; Connelly et al., 2020; Corbett et al., 2021; Nicolas et al., 2021). Therefore, the study of the relationship between the emergence of anxiety-like behaviors and incubation of cocaine craving in female rats is an important subject for future research.

## Conclusion

We studied the relationship between the emergence of anxiety-like states and incubation of cocaine craving after intermittent or continuous access cocaine self-administration. Our study provides no evidence that anxiety-like states contribute to incubation of cocaine craving. However, the interpretation of our negative data and their translational implications should be made with caution because we used a single anxiety-related test in male rats.

## Data Availability Statement

The raw data supporting the conclusions of this article will be made available by the authors, without undue reservation.

## Ethics Statement

The animal study was reviewed and approved by the Animal Care and Use Program (ACUC) of NIDA-IRP.

## Author Contributions

CN and TR carried out the experiments and performed the data analysis. CN, YS, and SI designed the study and wrote the manuscript. All authors critically reviewed the content and approved the final version before submission.

## Conflict of Interest

The authors declare that the research was conducted in the absence of any commercial or financial relationships that could be construed as a potential conflict of interest.

## Publisher’s Note

All claims expressed in this article are solely those of the authors and do not necessarily represent those of their affiliated organizations, or those of the publisher, the editors and the reviewers. Any product that may be evaluated in this article, or claim that may be made by its manufacturer, is not guaranteed or endorsed by the publisher.
