# Supplementary material for: Dissociation Between Incubation of Cocaine Craving and Anxiety-Related Behaviors After Continuous and Intermittent Access Self-Administration
Source: Front Neurosci. 2022 Feb 7;15:824741. doi: 10.3389/fnins.2021.824741 (PMC8859112; doi:10.3389/fnins.2021.824741)
Supplement: Supplementary file 1 [file Data_Sheet_1.pdf]

Supplementary Online Material

Dissociation of incubation of cocaine craving and anxiety-related behaviors after continuous and intermittent access self-administration

Table of content

Supplementary figure 1
Supplementary Table 1

Supplementary Figure 1.

Novelty-induced hypophagia **Defensive burying**

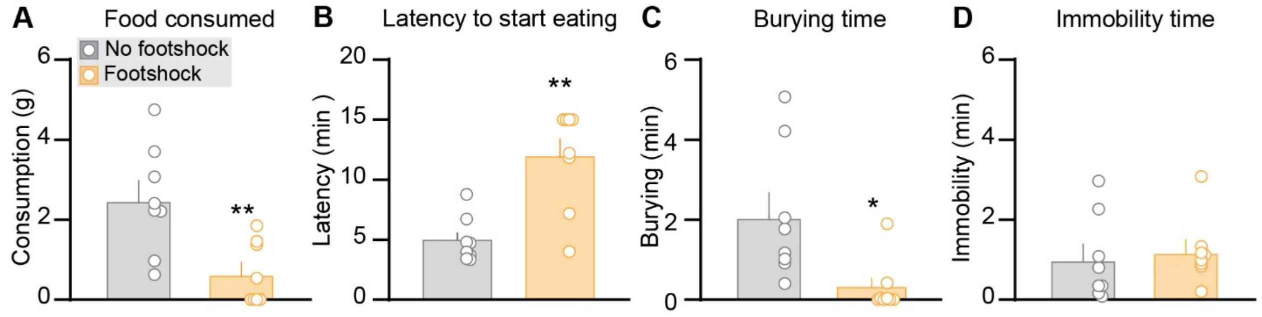

Intermittent footshock-induced anxiety-related behaviors in the novelty-induced hypophagia and defensive burying tests.

<u>Novelty-induced hypophagia.</u> **(A)** <u>Food consumed</u>: Mean ± SEM grams of food consumed (no footshock, n=8; intermittent footshock, n=8). **(B)** <u>Latency to start eating</u>: Mean ± SEM minutes to start (no footshock, n=8; intermittent footshock, n=8).

<u>Defensive burying.</u> **(C)** <u>Burying time</u>: Mean ± SEM time of probe burying per min (no footshock, n=8; intermittent footshock, n=8). **(D)** <u>Immobility time</u>. Mean ± SEM immobility time in minute (no footshock, n=8; intermittent footshock, n=8). Different from intermittent footshock * $p<0.05$ and ** $p<0.01$.

**Table S1.** Statistical analysis (SPSS GLM repeated-measures module)			
	**Factor Name**	**F-values**	**p-values**
Fig 1B<br>Cocaine intake (acquisition)	Schedule (between)<br>Session (within)<br>Group X Session	$F_{1,16}$=0.12<br>$F_{6,96}$=4.86<br>$F_{6,96}$=0.35	0.73<br>0.0002*<br>0.91
Fig 1B<br>Cocaine intake (training)	Group (between)<br>Session (within)<br>Group X Session	$F_{1,16}$=52.80<br>$F_{13,208}$=8.69<br>$F_{13,208}$=1.12	<0.0001*<br><0.0001*<br>0.35
Fig 1C<br>Frequency of intake (acquisition)	Group (between)<br>Session (within)<br>Group X Session	$F_{1,16}$=0.11<br>$F_{6,96}$=4.86<br>$F_{6,96}$=0.38	0.74<br>0.0002*<br>0.89
Fig 1C<br>Frequency of intake (training)	Group (between)<br>Session (within)<br>Group X Session	$F_{1,16}$=75.54<br>$F_{13,208}$=4.98<br>$F_{13,208}$=1.85	0.0001*<br>0.0001*<br>0.04*
Fig 1D<br>Relapse test	Group (between)<br>Abstinence day (within)<br>Lever (within)<br>Group X Abstinence day<br>Group X Lever<br>Abstinence day X Lever<br>Group X Abstinence day X Lever	$F_{1,16}$=16.39<br>$F_{1,16}$=33.15<br>$F_{1,16}$=38.84<br>$F_{1,16}$=0.03<br>$F_{1,16}$=1.26<br>$F_{1,16}$=37.60<br>$F_{1,16}$=0.98	0.0009*<br><0.0001*<br><0.0001*<br>0.86<br>0.28<br><0.0001*<br>0.34
Fig 1E<br>Progressive ratio (final ratio)	Group (between)	t_{15}=2.64	0.02*
Fig 1E<br>Progressive ratio (infusions)	Group (between)	t_{15}=2.47	0.03*
Fig 2A<br>Food consumed	Group (between)<br>Test order (between)<br>Abstinence day (within)<br>Group X Test order<br>Group X Abstinence day<br>Order X Abstinence day<br>Group X Test order X Abstinence day	$F_{2,23}$=1.25<br>$F_{1,23}$=0.71<br>$F_{1,23}$=43.68<br>$F_{2,23}$=0.57<br>$F_{2,23}$=0.35<br>$F_{1,23}$=0.48<br>$F_{2,23}$=0.44	0.31<br>0.41<br><0.0001*<br>0.58<br>0.71<br>0.49<br>0.65
Fig 2 B<br>Latency to eat	Group (between)<br>Test order (between)<br>Abstinence day (within)<br>Group X Test order<br>Group X Abstinence day<br>Test order X Abstinence day<br>Group X Test order X Abstinence day	$F_{2,23}$=0.74<br>$F_{1,23}$=0.65<br>$F_{1,23}$=6.75<br>$F_{2,23}$=2.53<br>$F_{2,23}$=0.27<br>$F_{1,23}$=0.55<br>$F_{2,23}$=0.59	0.49<br>0.43<br>0.02*<br>0.10<br>0.76<br>0.47<br>0.56
Fig 3A<br>Correlation food consumed and cocaine seeking day 1	Continuous access<br>Intermittent access	R^2=0.02<br>R^2=0.003	0.66<br>0.91
Fig 3B<br>Correlation food consumed and cocaine seeking day 21	Continuous access<br>Intermittent access	R^2=0.04<br>R^2=0.05	0.58<br>0.34
Fig 3C<br>Correlation food consumed and incubation scores	Continuous access<br>Intermittent access	R^2=0.17<br>R^2=0.16	0.21<br>0.37
Fig 4A<br>Correlation latency to eat and cocaine seeking day 1	Continuous access<br>Intermittent access	R^2=0.002<br>R^2=0.16	0.88<br>0.36
Fig 4B<br>Correlation latency to eat and cocaine seeking day 21	Continuous access<br>Intermittent access	R^2=0.009<br>R^2=0.01	0.78<br>0.80
Fig 4C Correlation latency to eat and incubation scores	Continuous access<br>Intermittent access	R^2=0.04<br>R^2=0.08	0.58<br>0.54
Supplementary Figure 1A<br>Food consumed	Footshock condition (between)	t_{14}=3.33	0.005*

Supplementary Figure 1B<br>Latency to eat	Footshock condition (between)	t_{14}=4.28	0.0008*
Supplementary Figure 1C<br>Burying time	Footshock condition (between)	t_{14}=2.79	0.01*
Supplementary Figure 1D<br>Immobility	Footshock condition (between)	t_{14}=0.41	0.69
